# Conservative Non-intervention Approach for Hemodynamically Significant Patent Ductus Arteriosus in Extremely Preterm Infants

**DOI:** 10.3389/fped.2020.605134

**Published:** 2020-12-23

**Authors:** Se In Sung, Yun Sil Chang, So Yoon Ahn, Heui Seung Jo, Misun Yang, Won Soon Park

**Affiliations:** Department of Pediatrics, Samsung Medical Center, Sungkyunkwan University School of Medicine, Seoul, South Korea

**Keywords:** patent ductus arteriosus, premature infants, extremely preterm infants, conservative management, non-intervention

## Abstract

While persistent patent ductus arteriosus (PDA) in preterm infants has been known to be associated with increased mortality and morbidities including bronchopulmonary dysplasia, and necrotizing enterocolitis, there is minimal evidence supporting their causal relationships, and most traditional medical and/or surgical treatments have failed to show improvements in these outcomes. As such, the pendulum has swung toward the conservative non-intervention approach for the management of persistent PDA during the last decade; however, the benefits and risks of this approach are unclear. In this mini review, we focused on whom, when, and how to apply the conservative non-intervention approach for persistent PDA, especially in extremely preterm infants.

## Introduction

Persistent patent ductus arteriosus (PDA) in premature infants has been known to be associated with increased mortality and morbidities including bronchopulmonary dysplasia (BPD), and necrotizing enterocolitis (NEC) (1–3); however, there is minimal evidence to support their causal relationship (3–5). Although it is traditional to manage PDA with nonsteroidal anti-inflammatory agents such as indomethacin, ibuprofen and recently acetaminophen (6), and/or surgical ligation, definite evidence supporting the benefits of these therapies over the conservative non-intervention approach is lacking (7–9), and they may even be associated with adverse effects (10–14). As such, the pendulum has swung toward the conservative non-intervention approach for managing hemodynamically significant (HS) PDA in preterm infants over the last decade (15, 16). However, the risks and benefits of the conservative non-intervention approach still remain unclear with variable outcomes showing no effect (17–19), reducing (16, 20) or increasing bronchopulmonary dysplasia (BPD) incidence, or death compared with traditional medical/surgical therapies (9, 21). These controversial outcomes of the conservative non-intervention approach may be attributable to confounding PDA treatment indications or disease severity (16, 17, 19), bias of non-independent sampling (16, 17), or lack of patient stratification according to gestational age (9, 16, 21). In this mini review, we focused on identifying those indicated for treatment, its optimal timing, and the method of applying the conservative non-intervention approach for HS PDA especially in extremely preterm infants (EPTs).

## Conservative Non-Intervention Approach

### Rationale for the Conservative Non-intervention Approach for HS PDA

The rationale of a mandatory closure approach with medical and/or surgical treatment for HS PDA is based on the deeply ingrained hypothesis that large left-to-right shunting via PDA directly causes increased mortality and morbidities (2, 22–25). The closure rate of HS PDA after treatment with cyclooxygenase inhibitors was inversely proportional to gestational age (GA), being as low as 29% in EPTs with GA of 23–26 weeks (20, 26–29); and ultimately, >72% received ductal ligation even with a more conservative approach for treating PDA in these infants not responding to medical treatment (20, 30). However, there is a possibility that the detrimental effects of surgical ligation may outweigh the benefits derived from PDA closure (11, 31, 32). Moreover, although prolonged exposure to large left-to-right shunting via HS PDA may increase the risk of mortality and morbidities (9, 33–37), no significant differences in mortality and morbidities such as NEC and IVH and even significantly lower incidences of BPD were observed with the conservative non-intervention approach compared with the mandatory closure approach in our previous retrospective study (20). In our recently conducted prospective double-blind randomized controlled trial (RCT) that directly compared the therapeutic efficacy of exclusive pharmacologic treatment with the standard dose of oral ibuprofen-mediated PDA closure and a non-intervention approach with few backup treatments (38), oral ibuprofen treatment significantly promoted the ductal closure rate at 1 week after randomization in the 27–30-week but not in the 23–26-week GA subgroup compared to the non-intervention approach, but the ultimate ductal closure rate and incidence of BPD/death were not significantly different between the two study groups. Recent studies have suggested that high-dose ibuprofen has better efficacy especially when used later, though data among micro-premature infants is lacking (39, 40). Considering the postnatal rise of serum creatinine levels indicative of low glomerular filtration rate due to immature nephrogenesis peaking at postnatal day 10 and 14 in EPTs at 23–24 and 25–26 weeks' gestation (41), standard rather than high dose oral ibuprofen therapy at first postnatal weeks was pharmacokinetically more ideal in the EPTs (40). The non-inferiority of non-intervention approach was partly attributable to the low response rate of standard-dose ibuprofen especially in EPTs at 23–26 weeks' gestation. However, considering the high ultimate treatment failure rate of 71 and 62% even with repeated doses of intravenous indomethacin and ibuprofen in the infants at 23–26 and 23–25 weeks' gestation (20, 26), our data of low response rate of oral ibuprofen for PDA closure in the infants at 23–26 but not at 27–30 weeks' gestation might be more attributable to younger gestational age of study population rather than to low therapeutic efficacy of oral ibuprofen therapy for closing PDA. Moreover, our data of 89–82% PDA closure rates before discharge in the ibuprofen and non-intervention arms with virtually no backup treatment, were much higher than the previously known overall PDA closure rate of 67% with medical treatment in premature infants (39). Our data showed a relatively lower 44% BPD/death rate in the non-intervention arm with virtually no backup treatment compared to that of PDA TOLERATE trial showing a 57% BPD/death rate with ligation rate of 12% in the conservative approach arm. Altogether, a current lack of definite evidence showing therapeutic benefits of traditional medical/surgical therapies over conservative treatment with supportive care endorse the conservative non-intervention approach allowing spontaneous closure as an alternative management option to the mandatory closure approach for HS PDA in EPTs (3, 4, 7, 20, 38, 42–44).

### Who Is Indicated for the Conservative Non-intervention Approach?

While a staging system assessing the magnitude of HS PDA based on clinical, biochemical, and echocardiographic criteria has been proposed (13, 45, 46), a clear definition of PDA shunt size and clinical illness severity eligible for the conservative non-intervention approach in preterm infants with persistent PDA is still lacking because of the paucity of available data for this approach (9, 11, 47–51). Moreover, practical difficulties exist in quantifying the shunt volume to distinguish which PDA are truly symptomatic (46). Discerning the hemodynamic significance of preterm PDA is challenging and should incorporate the echocardiographic measurements to assess the magnitude of ductal shunt volume, and the parameters reflecting the impact of shunt volume on end-organ systems. Therefore, the meaning of HS PDA may vary from one another in the clinical studies. Future studies about defining the hemodynamic significance of PDA should focus on the clinical outcomes rather association with the shunt volume (1). In the PDA-TOLERATE (PDA-TO Leave it alone or Respond And Treat Early) RCT (4), the enrollment criterion for HS PDA was defined as moderate-to-large PDA with a ductal size > 1.5 mm and predominant left-to-right shunt in EPTs (GA < 28 weeks) with respiratory failure requiring assisted respiratory support. However, this trial had significant selection bias as 40% of potentially eligible EPTs were not recruited, and received active treatment outside the PDA-TOLERATE trial because of “lack of physician equipoise”(52). Moreover, this trial was also confounded by a high backup rescue treatment rate of 48%, with 12% of patients receiving PDA ligation even in the conservative treatment arm (4). In our neonatal intensive care unit (NICU), we first introduced the conservative non-intervention approach in EPTs (GA < 27 weeks) who did not require early ventilator support regardless of PDA size (9, 47, 49). After confirming the tolerability of this approach without adverse effects, we switched the policy for the management of severe HS PDA (PDA size > 2 mm requiring mechanical ventilator support with symptoms/signs suggestive of PDA) in EPTs (GA = 23–26 weeks) from a mandatory PDA closure approach with indomethacin and/or surgical ligation to a conservative non-intervention approach without pharmacological/surgical treatments (20). Our results showed that the non-intervention approach was associated with less BPD compared with mandatory closure approach. Although backup medical/surgical treatment was available at the attending neonatologist's discretion, no EPT managed with the conservative non-intervention approach for HS PDA received backup treatment. However, the comparison of outcomes in the epoch-based study can be biased by the improvement of global care throughout the study period. Our data showing improved BPD rate without any significant differences in mortality or other morbidities between the two study periods might support the benefits of non-intervention over the mandatory closure, probably attributable to the avoidance of surgical ligation rather than to improved global care. In our recently conducted double-blind RCT (38), only EPTs with symptomatic PDA requiring respiratory support (average PDA size of 2.5 mm and left atrium/aorta ratio of 1.61 compatible with moderate-to-severe clinical and echocardiographic severity) were enrolled in the non-intervention arm, and received no backup treatment. Furthermore, as conservative non-intervention approach has become a standard treatment for HS PDA in EPT since 2012 (20), and all the EPTs not enrolled in our RCT were exclusively managed with conservative non-intervention approach with virtually no rescue treatment (38). Overall, these findings suggest that all EPTs, even those with the most severe HS PDA, could be indicated for management with the conservative non-intervention approach regardless of the clinical and echocardiographic severity.

### What Is the Optimal Timing for the Conservative Non-intervention Approach?

Determining the optimal timing of the mandatory closure approach with medical/surgical treatment or conservative non-intervention approach for managing HS PDA in EPTs is a critical issue in clinical settings. As the PDA may close spontaneously even in EPTs (19, 53–55), we avoided unnecessary treatment exposure in 37% of eligible patients by delaying their enrollment for approximately 1 week in our recently conducted RCT (38). However, there is a concern that delaying the pharmacologic PDA treatment up to the first postnatal week may increase mortality or morbidities (5, 52, 56, 57). In a prospective double-blind RCT of eligible infants <29 weeks' gestation, Kluckow et al. observed that early indomethacin treatment of a large PDA in patients under 12 h of age reduced pulmonary hemorrhage (PH) from 21 to 2%, IVH from 12.5 to 4.5%, and later backup treatment of PDA from 40 to 20%, but had no effect on the primary outcome of death or abnormal cranial ultrasound (55). In contrast, in our RCT, the incidences of IVH in oral ibuprofen and non-intervention arms were 3 and 6%, respectively; and there was no PH in both arms (38). These findings suggest that prophylaxis alone is not enough for better therapeutic outcome of HS PDA, and that just applying the conservative non-intervention approach alone also is not enough, but the accompanying meticulous neonatal intensive care is essential for the success of this approach (20, 38, 41, 54). The meticulous care starting from better delivery room management at birth includes judicious fluid restriction with high humidification to avoid volume overload and the ensuing congestive heart failure, ventilatory strategies to minimize pulmonary over-circulation including less oxygen use, and strict infection control practices, which will be discussed in detail later in section How to Apply the Conservative Non-intervention Approach? As the prevalence of HS PDA at 1 week after birth is inversely proportional to GA (93, 64, and 21% in EPTs with GA of 23–24, 25–26, and 27–28 weeks, respectively) (54), the lack of stratification according to GA may be the primary factor responsible for the controversial inconsistent outcomes of delaying PDA treatment until the first postnatal week (9, 21, 58). Collectively, these findings suggest that there is no critical therapeutic time window for the beneficial therapeutic efficacy of traditional pharmacologic/surgical treatment, and that the accompanying meticulous neonatal intensive care starting from birth is prerequisite for the success of the conservative non-intervention approach for HS PDA in EPTs (3, 20, 38, 41, 43, 44, 54).

Recently, neonatologist performed echocardiography (NPE) has been proposed as an instrumental diagnostic tool for early screening of HS PDA (59). However, as noted by the recommendation of the European Society for Pediatric Research and European Society for Neonatology (60), care should be taken against the indiscriminate application without considerable skill and experience. Frequent examination of the babies in the early critical period of life may have a hazardous effect on the patient safety; the lack of good repeatability of the echocardiographic measurements and the misinterpretation of those parameters can lead to overtreatment for PDA that can mostly close spontaneously without increasing neonatal morbidities. Further studies are needed to evidence the feasibility and the robustness of NPE.

### Natural Course of HS PDA With the Conservative Non-intervention Approach

Owing to the traditional mandatory closure approach of HS PDA with medical and/or surgical treatment at the earliest time based on the old dogma that adverse outcomes are proportional to the magnitude and duration of left-to-right ductal shunting (2, 61), and the high backup treatment rates even with the conservative non-intervention approach for HS PDA (4, 52, 55), few studies have chronologically monitored the natural history of HS PDA in EPTs. Rolland et al. observed a 73% spontaneous closure rate in EPTs with <28 weeks' gestation without specific treatment aimed to close PDA (62). Semberova et al. reported an 85% spontaneous PDA closure rate before hospital discharge in very low birth weight infants who received truly conservative PDA management (63). In our previous study, only 3% of EPTs with GA of 23–28 weeks with HS PDA solely managed with a conservative non-interventional approach patients were discharged home without ductal closure, 2% had spontaneous closure on outpatient follow-up, and 1% received device closure (54). Overall, these findings suggest that spontaneous closure of HS PDA could be achieved solely with conservative non-interventional management even in EPTs near the limit of viability, and that exposure to the risks of medical/surgical therapeutic interventions targeted for ductal closure may not be warranted in these infants.

Despite the current lack of evidence supporting the causal relationships between a large left-to-right shunt via HS PDA and mortality/morbidities, significant associations between large PDA shunt volumes and morbidities such as BPD were observed in some studies (1, 13, 45, 64). In contrast, the presence of HS PDA was not associated with increased mortality or morbidities such as severe IVH, BPD, NEC, acute kidney injury, retinopathy of prematurity, and sepsis compared with EPTs without HS PDA (41, 54, 65). Besides the magnitude, the prolonged duration of large left-to-right shunting via PDA may be an important cause of mortality and/or morbidities. Shena et al. reported that each week of HS PDA increased the risk of developing BPD in EPTs with <28 weeks' gestation by 1.7 times, and a prolonged PDA with later surgical ligation (33 vs. 23 days) was associated with increased death or BPD (51). In contrast, we previously showed a significantly lower incidence of BPD and comparable mortality despite significantly delayed closure of HS PDA at postnatal day 44 with a conservative non-intervention approach compared with earlier mandatory closure with medical/surgical treatments at postnatal day 13 in EPTs with GA of 23–26 weeks (20). Moreover, while the mean closure time of HS PDA was inversely proportional to GA (P53, P41, and P36 in EPTs with GA of 23–24, 25–26, and 27–28 weeks, respectively), a prolonged duration per week of HS PDA with a conservative non-intervention approach was not associated with increased mortality and/or morbidities in multivariate analyses adjusted for GA and birth weight (54). Despite the limitation of the previous studies including the retrospective nature of the study design, the seeming association between the presence and prolonged duration of HS PDA and mortality/morbidities may be primarily attributable to immaturity itself, and our data showing a favorable outcome of the conservative non-interventional approach for HS PDA in EPTs at least support the safety and feasibility of this approach over traditional medical/surgical treatments for managing HS PDA in EPTs (20).

### How to Apply the Conservative Non-intervention Approach?

As excessive fluid intake increased the risk of HS PDA and the ensuing congestive heart failure (66–68), the reasons why prolonged exposure to HS PDA with the conservative non-intervention approach did not increase mortality/morbidities in our previous studies may be primarily attributable to judicious fluid restriction (20, 30, 38, 54). In order to achieve judicious fluid restriction, restricting the first-day fluid intake to as low as 60 ml/kg/day even in peri-viable EPTs with GA of 23–24 weeks must be accompanied by meticulous neonatal intensive care including better delivery room management with extreme caution of fragile skin care, minimal handling, and high humidification during the first several postnatal days (69, 70). We individualized and adjusted the daily target fluid volume for each EPT after assessment of the volume status by monitoring the urine output and specific gravity, body weight, serum electrolytes, and estimating the insensible water loss (41). Due to the greater and delayed peak of serum creatinine levels during the first few postnatal weeks in EPTs, indicative of low glomerular filtration, we tried to avoid fluid overload by maintaining a low fluid intake (<116 ml/kg/day) during the first 4 postnatal weeks without restricting caloric intake or increasing the incidence of electrolyte and renal dysfunctions (20, 41, 54). In our previous retrospective study in which we applied the same fluid policy, the incidence of hypernatremia (>150 mEq/dL sodium) was 41, 21, and 15% in 23, 24, and ≥25 weeks' gestation infants (71). Taken together, these findings suggest that merely applying conservative management for HS PDA is not sufficient to achieve better clinical outcomes, and meticulous NICU care, including judicious fluid restriction, is a prerequisite for successful clinical translation of the non-intervention approach to manage HS PDA in EPTs.

While Liebowitz and Clyman reported a high incidence of HS PDA (67%) at the first postnatal week in EPTs (mean GA = 26 weeks) with a first-day fluid intake of 166 ml/kg/day, a much lower incidence (57%) was reported in EPTs (mean GA = 25 weeks) with a first-day fluid intake of 67 ml/kg/day in our previous study (21, 54). Moreover, while Semberova et al. reported >30% of infants with birth weights of 750–999 g taking liberal fluid intake were discharged home with open ductus, we observed only <10% of EPTs (GA of 23–26 weeks) with restricted fluid intake of 107–115 ml/kg/day between P7–28 discharged home with open ductus in our previous studies (19, 20, 38, 54). Collectively, these findings suggest that judicious fluid restriction to avoid excessive fluid intake may be essential to reduce the prevalence of HS PDA and promote its earlier closure, thereby reducing the associated mortality/morbidities (66).

Although *pro re nata* diuretics were more frequently used with the conservative non-intervention approach than with the mandatory closure approach in our previous study (20), their therapeutic efficacy has not been proven in EPTs with immature tubular function (41), and furosemide may even promote continued patency of PDA (72). Inotropic support was used more frequently with the mandatory closure approach than with the conservative non-intervention approach in our previous study (20). High frequency ventilation may be effective for reducing pulmonary blood flow via PDA. Further studies are necessary to further clarify these controversial issues.

As the risk of developing BPD in EPTs with HS PDA increased substantially with concurrent bacterial sepsis (73), the implementation of clinical strategies to reduce nosocomial sepsis including reduced use of invasive procedures and antibiotics may be important for ameliorating the potential adverse effects associated with HS PDA (74, 75).

## Discussion

Recent evidences show the feasibility and the efficacy of more conservative approach in the management of PDA compared to mandatory treatment approach, resulting in lower PDA ligation rates and similar neonatal outcomes (4, 7, 30, 38). Accordingly, there was an increasing worldwide trend toward conservative management for HS PDA in preterm infants. The recently published trial suggested that the conservative non-intervention approach with virtually no backup traditional medical/surgical treatments could be indicated even for the most severe cases of HS PDAs regardless of its clinical and echocardiographic severity, and showed that almost all HS PDAs eventually closed spontaneously without increased mortality/morbidities even in the peri-viable EPTs (38). However, just applying conservative nonintervention approach is not enough, and meticulous neonatal intensive care including better delivery room management with extreme caution on skin care, judicious fluid restriction starting from the first day until at least 4 postnatal weeks along with minimal handling and high humidification during first several postnatal days, better infection control strategies to prevent sepsis, and less invasive assisted ventilator strategies including less use of oxygen are essential for the success of the conservative non-intervention approach for HS PDA in EPTs (54, 68, 69, 71, 74). Our findings also support the need for future studies to reexamine and better clarify the pros and cons of the conservative non-intervention approach for HS PDA in EPTs.

## Author Contributions

SS drafted and wrote the manuscript. YC and SA conceived, reviewed, and revised the manuscript. HJ and MY were involved in literature search and revised the manuscript. WP conceived, drafted, and critically revised the manuscript. All authors contributed to the article and approved the submitted version.

## Conflict of Interest

The authors declare that the research was conducted in the absence of any commercial or financial relationships that could be construed as a potential conflict of interest.
